# Allogeneic hematopoietic stem cell transplantation can improve the prognosis of high-risk pediatric t(8;21) acute myeloid leukemia in first remission based on MRD-guided treatment

**DOI:** 10.1186/s12885-020-07043-5

**Published:** 2020-06-15

**Authors:** Guan-hua Hu, Yi-fei Cheng, Ai-dong Lu, Yu Wang, Ying-xi Zuo, Chen-hua Yan, Jun Wu, Yu-qian Sun, Pan Suo, Yu-hong Chen, Huan Chen, Yue-ping Jia, Kai-yan Liu, Wei Han, Lan-ping Xu, Le-ping Zhang, Xiao-jun Huang

**Affiliations:** 1 Department of Pediatrics, Peking University People’s Hospital, Peking University, No. 11, Xizhimen South Street, Xicheng District, Beijing, 100044 China; 2 Peking University People’s Hospital, Peking University Institute of Hematology, National Clinical Research Center for Hematologic Disease, Beijing Key Laboratory of Hematopoietic Stem Cell Transplantation, Peking-Tsinghua Center for Life Science, Research Unit of Key Technique for Diagnosis and Treatment of Hematologic Malignancies, Chinese Academic of Medical Sciences, No.11, Xizhimen South Street, Xicheng District, Beijing, 100044 China

**Keywords:** *RUNX1-RUNX1T1* transcript levels, Childhood acute myeloid leukemia, Allogeneic hematopoietic stem cell transplantation, Relapse

## Abstract

**Background:**

Pediatric acute myeloid leukemia (AML) with t(8;21) (q22;q22) is classified as a low-risk group. However, relapse is still the main factor affecting survival. We aimed to investigate the effect of allogeneic hematopoietic stem cell transplantation (allo-HSCT) on reducing recurrence and improving the survival of high-risk pediatric t(8;21) AML based on minimal residual disease (MRD)-guided treatment, and to further explore the prognostic factors to guide risk stratification treatment and identify who will benefit from allo-HSCT.

**Methods:**

Overall, 129 newly diagnosed pediatric t(8;21) AML patients were included in this study. Patients were divided into high-risk and low-risk group according to *RUNX1-RUNX1T1* transcript levels after 2 cycles of consolidation chemotherapy. High-risk patients were divided into HSCT group and chemotherapy group according to their treatment choices. The characteristics and outcomes of 125 patients were analyzed.

**Results:**

For high-risk patients, allo-HSCT could improve 5-year relapse-free survival (RFS) rate compared to chemotherapy (87.4% vs. 61.9%; *P* = 0.026). Five-year overall survival (OS) rate in high-risk HSCT group had a trend for better than that in high-risk chemotherapy group (82.8% vs. 71.4%; *P* = 0.260). The 5-year RFS rate of patients with a c-KIT mutation in high-risk HSCT group had a trend for better than that of patients with a c-KIT mutation in high-risk chemotherapy group (82.9% vs. 75%; *P* = 0.400). Extramedullary infiltration (EI) at diagnosis was associated with a high cumulative incidence of relapse for high-risk patients (50% vs. 18.4%; *P* = 0.004); allo-HSCT can improve the RFS (*P* = 0.009).

**Conclusions:**

allo-HSCT can improve the prognosis of high-risk pediatric t(8;21) AML based on MRD-guided treatment. Patients with a c-KIT mutation may benefit from allo-HSCT. EI is an independent prognostic factor for high-risk patients and allo-HSCT can improve the prognosis.

## Background

Translocation (8;21) (q22;q22) or *RUNX1-RUNX1T1* rearrangement comprises 10–15% of pediatric acute myeloid leukemia (AML) and is known to have a favorable outcome [1]. However, approximately 30% of patients ultimately relapse and even with allogeneic hematopoietic stem cell transplantation (allo-HSCT), the prognosis of patients who relapse remains poor [2]. Therefore, the present study aimed to establish how to identify high-risk patients and determine stratified treatment to reduce disease recurrence and improve survival.

Cytogenetics is an important standard for risk stratification in pediatric AML [3, 4]. Minimal residual disease (MRD) monitoring based on cytogenetic stratification is useful for assessing susceptibility to chemotherapy and making risk stratification more accurate and instructive. St Jude Children’s Research Hospital recently reported a trial of an MRD-directed risk stratification strategy to successfully improve the outcomes of high-risk pediatric patients with AML [5]. For pediatric t(8;21) AML, many clinical trials have confirmed that monitoring *RUNX1-RUNX1T1* transcript levels can effectively predict relapses and direct clinical interventions [6, 7]. However, it is currently unclear how the prognosis of high-risk pediatric t(8;21) AML with allo-HSCT based on MRD-guided therapy can be improved due to the low incidence of pediatric t(8;21) AML and fewer patients undergoing allo-HSCT.

In this study, we performed MRD-guided treatment on 125 patients and demonstrated that allo-HSCT can improve the prognosis of high-risk t(8;21) patients and analyzed the effect of other risk factors affecting the prognosis.

## Methods

### Patients

**Fig. 1:**
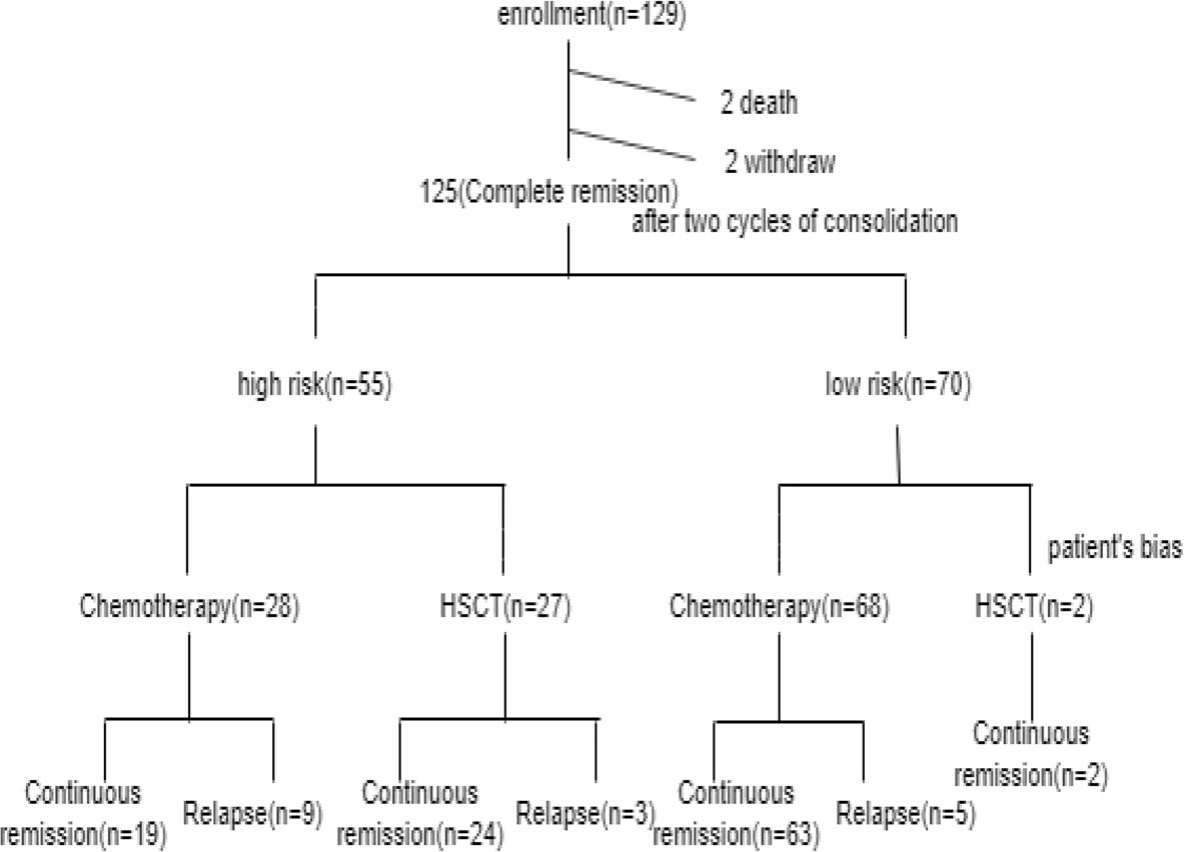
Trial design and patient accrual flowchart

Overall, 129 pediatric t(8;21) AML patients were enrolled between January 2011 and December 2017. The following inclusion criteria was applied: (1) 1 to 16 years old; (2) newly diagnosed with t(8;21) and/or *RUNX1/RUNX1T1* transcripts; and (3) achieved complete remission (CR) after 2 cycles of induction. Figure 1 presents the treatment scheme. Each patient’s parent or legal guardian signed an informed consent for chemotherapy and/or allo-HSCT. The study was approved by the Ethics Committee of Peking University People’s Hospital.

### MRD monitoring and c-KIT mutation screening

Bone marrow samples were collected at the time of diagnosis, before every cycle of chemotherapy, and then at 3-month intervals for 2 years, and at 6-month intervals for another 2 years. Real-time quantitative reverse transcription polymerase chain reaction (RT-PCR) was used to quantitatively detect the level of *RUNX1/RUNX1T1* transcripts. A direct sequencing method was used to screen for c-KIT mutations.

### Treatment response assessment and risk groups

CR was defined as the presence of < 5% blasts in bone marrow, an absolute neutrophil count > 1 × 10^9^/L, a platelet count > 100 × 10^9^/L, with no red cell transfusions and the absence of extramedullary disease. The recurrence of ≥5% bone marrow blasts and/or the development of extramedullary disease was defined as a relapse. After the second consolidation therapy, patients with *RUNX1-RUNX1T1* transcript levels > 0.05% were defined as high-risk. Patients with *RUNX1-RUNX1T1* transcript levels dropping to ≤0.05% after the second consolidation therapy were assigned to the low-risk group.

### Treatment protocols

Induction chemotherapy included cytarabine at 150 mg/m^2^ for 7 days (continuous infusion for day 1–2, and twice a day, 3 h for each infusion for day 3–7) in combination with anthracycline (idarubicin at 10 mg/m^2^ for 2 days) and etoposide at 100 mg/m^2^ for 3 days. Consolidation chemotherapy began after two induction cycles. Consolidation was composed of three regimens. Regimen 1: cytarabine (Ara-c 2 g/m^2^ for 4 days) with anthracycline (idarubicin at 10 mg/m^2^ for 2 days). Regimen 2: Harringtonine at 3 mg/m^2^ for 7 days with cytarabine at 150 mg/m^2^ for 7 days. Regimen 3: cytarabine at 150 mg/m^2^ for 7 days (continuous infusion for day 1–2, and twice a day, 3 h for each infusion for day 3–7) in combination with anthracycline (idarubicin at 10 mg/m^2^ for 2 days) and etoposide at 100 mg/m^2^ for 3 days. Alternate use of the three regimens was recommended for a total of 12–18 months. From October 2014, patients with c-KIT mutations were given tyrosine kinase inhibitor (TKI) drugs during chemotherapy intermission. For patients with a c-KIT D816V mutation or with CNSL, oral Dasatinib (50-70 mg/m^2^/d) was given, remaining patients with a c-KIT mutation were taken with oral Imatinib (270-340 mg/m^2^/d). TKIs were given from the beginning of the second course of chemotherapy to the end of chemotherapy. High-risk patients were recommended for allo-HSCT. Conditioning regimens were administered as previously described [8, 9]. Patients who received an HLA-mismatched HSCT received a regimen including cytarabine (4 g/m2/day, i.v.) on days − 10 and − 9, busulfan (BU) (3.2 mg/kg/day, i.v.) on days − 8 to − 6, cyclophosphamide (Cy) (1.8 g/m2/day, i.v.) on days − 5 and − 4, semustine (250 mg/m2, p.o.) on day − 3, and ATG (2.5 mg/kg/day, i.v.) from days − 5 to − 2. Patients who received an HLA-identical HSCT were treated with a regimen without ATG, which was identical to that of haploidentical HSCT recipients. All patients received aGVHD prophylaxis consisting of cyclosporine A, mycophenolate mofetil, and a short-term methotrexate regimen.

### Statistical methods

Relapse-free survival (RFS) was defined as the time between remission and relapse or death. Overall survival (OS) was defined as the time between diagnosis and death or the last follow-up. SPSS23.0 (SPSS Inc., Chicago, IL, USA) was used for data analysis. The Kaplan-Meier method was used to analyze RFS and OS, which was then analyzed with the log-rank sum test. The X^2^ test was performed to compare rates between groups. The Mann-Whitney or Wald-Wolfowitz test was performed to analyze significance of differences between continuous variables. Receiver operating characteristic (ROC) curve analysis was done to define the value with the highest sensitivity and specificity for predicting an event. Cox regression was performed to analyze factors that may affect RFS. *P* value < 0.05 was considered statistically significant.

## Results

### Patient characteristics

In this study, 129 newly diagnosed pediatric t(8;21) AML patients were enrolled. Four patients were excluded due to their death before 2 cycles of consolidation chemotherapy (*n* = 2) and withdrawal (*n* = 2). The remaining 125 patients were divided into a low-risk group (*n* = 70) and a high-risk group (*n* = 55), according to the *RUNX1-RUNX1T1* transcript levels after 2 cycles of consolidation chemotherapy. High-risk patients with *RUNX1-RUNX1T1* transcript levels > 0.05 after the completion of 2 cycles of consolidation chemotherapy were automatically divided into the HSCT group (*n* = 27) and chemotherapy group (*n* = 28) according to the parents’ wish, availability of donors, and economic conditions. In the high-risk HSCT group, twenty-five patients received haploidentical hematopoietic stem cell transplantation (haplo-HSCT) and two patients received HLA-matched sibling donor HSCT. General information, cytogenetic characteristics, and treatment responses for each group are summarized in Table 1. There were no significant differences between the high-risk chemotherapy group and high-risk HSCT group except age due to the choice bias caused by parents of younger patients who believe that the risk of transplantation is too high to pursue.

**Table 1:** Characteristics of enrolled patients

Characteristics	High-risk			Low-risk
	High-risk chemotherapy	High-risk HSCT	*P*	Total
Age (years)	9.5 ± 3.3	10.4 ± 4.3	0.036	8.7 ± 3.6
Gender (male/female)	14/14	18/9	0.072	38/32
WBC (10^9^)	25.6 ± 19.1	22.7 ± 26.4	0.697	16.8 ± 15.8
EI (%)	25	18.5	0.561	21.4
c-KIT mutation (%)	42.8	33.3	0.094	27.1
MRD after induction (%)	4.2 ± 9.7	3.1 ± 5.1	0.269	1.9 ± 1.2
MRD after the first consolidation (%)	1.4 ± 2.9	2.1 ± 3.4	0.226	0.08 ± 0.14
MRD after the second consolidation (%)	0.54 ± 1.0	0.9 ± 1.0	0.365	0.01 ± 0.01

*Abbreviations*: *EI* extramedullary infiltration, *HSCT* hematopoietic stem cell transplantation, *MRD* minimal residual disease, *WBC* white blood cell

### Patient outcomes

The median follow-up time was 46 months (10–96 months) in surviving patients. Of the 125 patients analyzed, 12 died (10 of relapse and 2 of treatment-related mortality) and 113 survived, 17 patients relapsed. For overall patients, the cumulative incidence of relapse (CIR) was 17.8%. The RFS and OS rates were 82.2 and 86%, respectively. Patients in high-risk HSCT group achieved neutrophil engraftment at a median time of 13 days (range, 10–38 days), platelet engraftment at a median time of 16 days (range, 7–47 days). At day + 100, the cumulative rates of grades II-IV aGVHD were 31.8% (95% CI, 20.1–48.9%), the cumulative rates of grades III-IV aGVHD were 14.6% (95% CI, 5.2–21.4%). The 3-year cumulative rates of moderate to severe cGVHD were 19.9% (95% CI, 8.3–25.8%), the 3-year cumulative rates of severe cGVHD were 19.8% (95% CI, 6.7–22.2%). One patient dead of non-relapse mortality in high-risk HSCT group.

### *RUNX1-RUNX1T1* transcript levels> 0.05% after second consolidation chemotherapy can effectively predict relapse

**Fig. 2:**
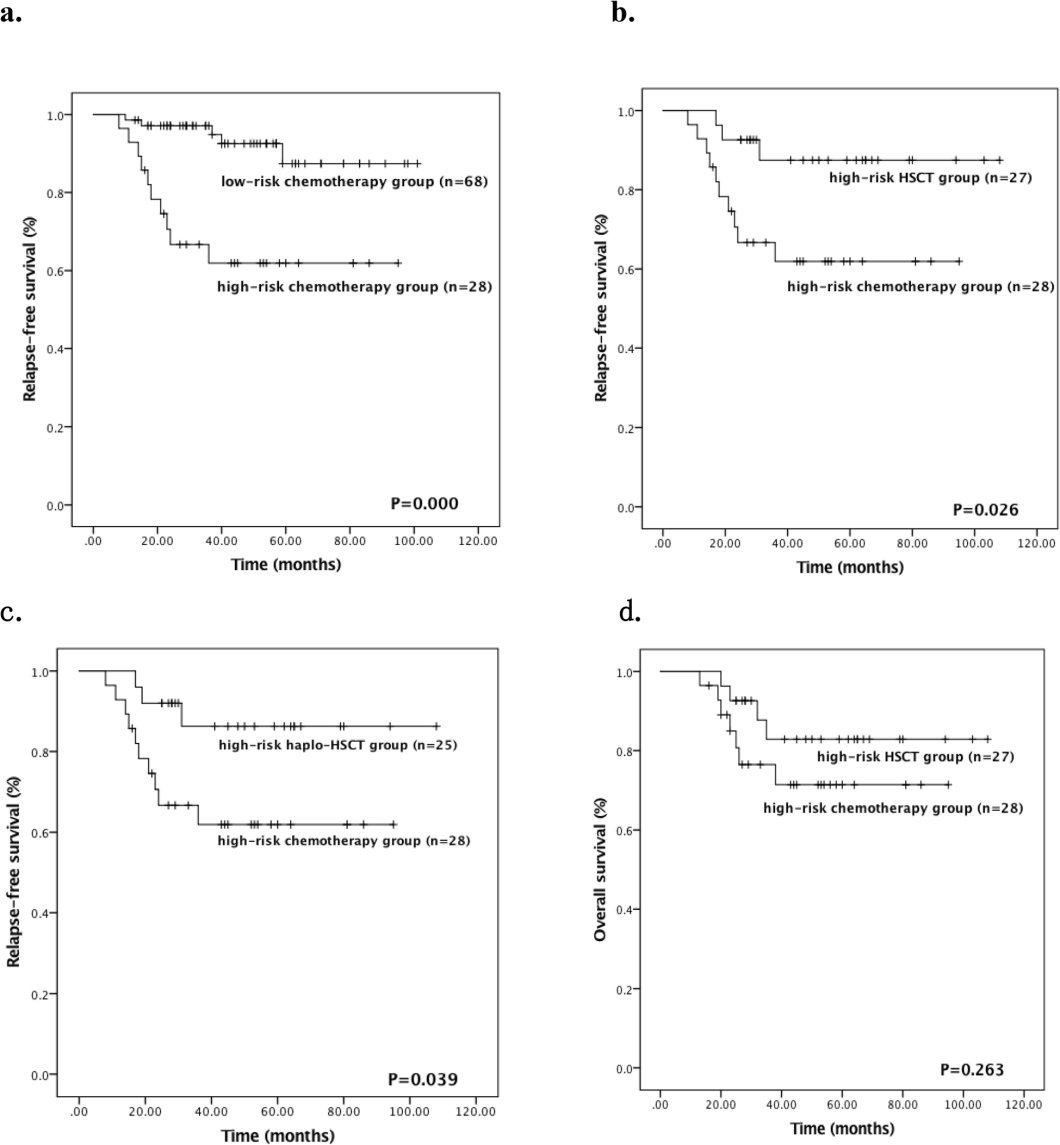
Kaplan-Meier Survival Curves Showing. **a** RFS in low-risk chemotherapy group and high-risk chemotherapy group, **b** RFS in high-risk HSCT group and high-risk chemotherapy group, **c** RFS in high-risk haplo-HSCT group and high-risk chemotherapy group, **d** OS in high-risk HSCT group and high-risk chemotherapy group. Abbreviations: haplo-HSCT, haploidentical hematopoietic stem cell transplantation; HSCT, hematopoietic stem cell transplantation; OS, overall survival; RFS, relapse-free survival

ROC analysis showed that a cutoff level of 0.05% in *RUNX1/RUNX1T1* transcripts level after two courses of consolidation chemotherapy significantly predicted an event (*P* = 0.030, the area under curve 0.660, sensitivity 88.9%, specificity 56.1%). A level significantly predicting an event could not be determined by ROC analysis after inducement chemotherapy and one course of consolidation chemotherapy. The survival of patients who received chemotherapy-based consolidation between the high-risk and low-risk groups was retrospectively analyzed. Patients with *RUNX1-RUNX1T1* transcripts ≤0.05% after the second consolidation had a significantly better 5-year RFS rate than those with> 0.05% (86.5% [95% CI, 86–99.8%]) vs. 61.9% [95% CI, 51.96–80.41%]; *P* = 0.000, Fig. 2a).

### Relapse-free survival

#### Allogeneic hematopoietic stem cell transplantation can improve RFS in the high-risk group

As shown in Fig. 2a, 5-year RFS rate was significantly better in the high-risk HSCT group than in the high-risk chemotherapy group (87.4% [95% CI, 86.0–108.7%]) vs. 61.9% [95% CI, 51.9–80.4%]; *P* = 0.026, Fig. 2b). Patients in high-risk HSCT group had comparable 5-year RFS rate with patients in low-risk group (87.4% [95% CI, 86.0–108.7%]) vs. 87.4% [95% CI, 86.9–99.8%]; *P* = 0.643).

In this study, 25 (92.5%) of patients in the high-risk HSCT group received haplo-HSCT and patients who received haplo-HSCT had significantly better 5-year RFS rate compared to patients who received chemotherapy-based consolidation (86.3% [95% CI, 84.0–108.7%]) vs. 61.9% [95% CI, 51.9–80.4%]; *P* = 0.039, Fig. 2c).

#### Outcomes of high-risk patients with c-KIT mutations

Among patients in high-risk group, twenty-one had c-KIT mutations detected at diagnosis. Twelve of the 21 patients were included in the chemotherapy group and the remaining nine were in the HSCT group. For high-risk patients with c-KIT mutations, HSCT had a potential to improve 5-year RFS rate (82.9% [95% CI, 59.3–84.8%]) vs. 75% [95% CI, 49.3–85.6%]; *P* = 0.400).

#### Outcomes of high-risk patients with EI

Among the high-risk chemotherapy group, seven patients had EI at diagnosis, including three orbital, one intracranial, one mandibular mass, one spine and one with orbital and lumbar spine infiltration, and six of these patients relapsed. One extramedullary relapsed first and the bone marrow relapsed 2 months later. In the high-risk HSCT group, five patients had EI at diagnosis, including two orbital, one scalp mass, one lumbar spine, and one with multiple vertebrae infiltration, and all of these patients were at remission after HSCT. RFS was significantly better in high-risk HSCT patients with EI (*n* = 7) than in high-risk chemotherapy patients with EI (*n* = 5, *P* = 0.006).

### Relapse

This study analyzed 125 patients, 17 of them relapsed, five relapses occurred in the low-risk group, twelve relapses occurred in the high-risk group. Notably, nine patients relapsed in high-risk chemotherapy group, six of them had EI at onset. Multivariate analysis of relapse-related factors in high-risk group showed that with EI at onset (HR 4.750, 95% CI: 1.537–14.678; *P* = 0.007) and non-HSCT (HR 0.238, 95% CI: 0.064–0.883; *P* = 0.032) were the independent risk factors for poor RFS (Table 2).

**Table 2:** Multivariate analysis of relapse-related factors among high-risk t(8;21) AML

Covariate	HR	95% CI	*P*
EI (with vs. without)	4.750	1.537–14.678	0.007
Treatment (HSCT vs. chemotherapy)	0.238	0.064–0.883	0.032
Age (≥10 years vs. < 10 years)^a^	0.451	0.143–1.423	0.174
WBC (≥20 × 10^9^ /L vs. < 20 × 10^9^)^a^	NS	NS	NS
c-KIT mutations (with vs. without)	NS	NS	NS
CD56 (positive vs. negative)	NS	NS	NS
CR after first inducement (yes vs. no)	NS	NS	NS

*Abbreviations*: *CI* confidence interval, *CR* complete remission, *EI* extramedullary infiltration, *HR* hazard ratio, *HSCT* hematopoietic stem cell transplantation, *WBC* white blood cell

^a^Cutoff based on median values

The average recurrence time in the high-risk group was 16.6 ± 7.34 months, and in the low-risk group was 28.86 ± 18.8 months. The recurrence time in the low-risk group was significantly latter than in the high-risk group (*P* = 0.011). 50% of relapses occurred after 3 years of treatment in low-risk group, while all the relapses occurred within 3 years of treatment in high-risk group.

### OS

The 5-year OS rate of overall patients was 86%. For the high-risk group, eleven patients died, four patients were in high-risk HSCT group, three patients died of relapse and one patient died of bronchiolitis obliterans syndrome. For the high-risk chemo group, a total of seven patients died of relapse. The 5-year OS rate in high-risk HSCT group had a trend to be better than that in high-risk chemotherapy group (82.8% [95% CI, 78.6–101.7%]) vs. 71.4% [95% CI, 62.09–87.6%]; *P* = 0.26, Fig. 2d). For the low-risk group, four patients died of relapse and the 5-year OS rate was 93.3%.

## Discussion

Patients with t(8;21) (q22; q22) or *RUNX1-RUNX1T1* rearrangement were classified as a low-risk and accounted for 10–15% of pediatric AML [1]. However, relapse is currently the main factor affecting the survival of pediatric t(8;21) AML patients, which is a problem that needs addressing. Many studies have demonstrated that MRD monitoring using RQ-PCR can effectively identify patients with higher risk of relapse [6, 10, 11], however, the most powerful timing and checkpoints for MRD monitoring were unclear. John A. et al. demonstrated that after course 3, the 2 most prognostic factors for relapse risk were 4 log reduction in BM and BM copy number > 500 [7]. ZHU et al. believed that MRD status after second consolidation may be the best timing [12]. ROC analysis in the current study showed that *RUNX1/RUNX1T1* transcripts level > 0.05% after two courses of consolidation chemotherapy significantly predicted an event, which was used as a dividing line between the low- and high-risk group, the high-risk patients had a high CIR compared to low-risk patients.

The antileukemic effect of HSCT has been established in multiple studies [13, 14]. However, due to the high rates of HSCT-related mortality and morbidity, HSCT is recommended for pediatric patients with high-risk AML. Therefore, this study is the first to explore the effect of HSCT for pediatric t (8; 21) patients. Based on MRD-guided risk stratification treatment, we demonstrated that allo-HSCT could significantly improve the RFS for high-risk t(8;21) AML. Meanwhile, high-risk HSCT patients has similar outcomes as low-risk patients, which means HSCT can negate the adverse effect of high transcripts number. In this study, 50% of low risk relapse patients relapsed after 3 years of treatment, while all high-risk groups relapsed within 3 years of treatment. We believe that relapse was more related to the fact that chemotherapy resistance and MRD uncleared completely for high-risk patients. This is may be the main reason for HSCT, which thoroughly cleared the residual leukemia and effectively reduced the recurrence rate for high-risk patients. For patients in low-risk group, relapse is more related to mechanisms such as clonal evolution [15], some trials have confirmed that those who had different cytogenetics at relapse had significantly improved survival after transplantation [16]. In this study, two patients of the low-risk group relapsed after 3 years of treatment and t(8; 21) disappeared after relapse. They received allo-HSCT as their salvage treatment after relapse and are currently at continuous remission.

Regarding OS, allo-HSCT had a trend to improve the OS for high-risk patients (82.8% vs. 71.4%; *P* = 0.26). This is consistent with the result of a previous study [17]. Whether the positive effect of HSCT in CR1 can be replaced by salvage-transplant after relapse was considered. Many clinical trials have confirmed that although some patients who have relapsed can survive through salvage therapy, the OS% is unsatisfactory and significantly lower than that of CR1 patients [18, 19]. Meanwhile, MRD levels before transplantation can predict the recurrence rate after transplantation [20, 21]. Therefore, HSCT is still necessary for some high-risk patients in CR1 to improve prognosis, keeping balance between the reducing risk of relapse and reducing transplant-related mortality to improve OS relies on precise risk stratification to guide treatment.

The prognostic significance of c-KIT mutations in pediatric t(8;21) AML is controversial. Some researchers believed that c-KIT mutations has no significance for pediatric AML, which point is different from adults [22, 23]. Some studies demonstrated that c-KIT mutation was a risk factor for pediatric t(8;21) AML, and c-KIT mutation was used as an indicator for transplantation [24, 25]. In this study, HSCT had a potential to improve the prognosis of high-risk patients with c-KIT mutation. However, the limited sample and part of patients were received TKIs, which may have affected the results.

Seven patients had EI at diagnosis in high-risk chemotherapy group, six of them relapsed. Multivariate analysis showed that EI was an independent risk factor for high-risk patients. In the high-risk HSCT group, five patients with EI at diagnosis experienced no recurrence after allo-HSCT, therefore, allo-HSCT may improve the prognosis of patients with EI. Studies regarding the significance of EI on pediatric AML are few and conflicting, even for t(8;21) AML which is the most closely related to EI. The Catholic University of Korea analyzed the characteristics and outcomes of 40 patients who were diagnosed with and treated for *RUNX1-RUNX1T1* (+) AML. They demonstrated that the presence of myeloid sarcoma type EI at diagnosis may predict the risk of relapse [26], which is consistent with our results. However, studies on the effect of allo-HSCT on pediatric t(8; 21) with EI are not available.

There are some limitations in this study. Firstly, this was a nonrandomization controlled trial which was a source of bias. However, recruiting large-scale numbers of patients for randomized trials is difficult and unrealistic for pediatric t(8;21) AML with 10% incidence, and there are no such studies currently in progress. Secondly, part of patients were received with TKIs which may affect the results of this study to some extent. Thirdly, the limited samples in each group was also a limitation of the study.

## Conclusion

We suggested that allo-HSCT may improve the prognosis of high-risk pediatric t(8;21) AML based on MRD-guided treatment. Patients with c-KIT mutation may benefit from allo-HSCT. Patients with EI at onset needed to monitor the condition of residual bone marrow leukemia and extramedullary lesions closely as these patients have higher recurrence rates and allo-HSCT may improve their prognosis.

## Data Availability

The datasets supporting the conclusions of this article are included within the article.

## Abbreviations

allo-HSCT: Allogeneic hematopoietic stem cell transplantation
AML: Acute myeloid leukemia
CI: Confidence interval
CIR: Cumulative incidence of relapse
CR: Complete remission
haplo-HSCT: Haploidentical hematopoietic stem cell transplantation
RFS: Relapse-free survival
EI: Extramedullary infiltration
MRD: Minimal residual disease
OS: Overall survival
RQ-PCR: Real-time quantitative PCR

## References

[1] Harrison CJ, Hills RK, Moorman AV, Grimwade DJ, Hann I, Webb DK (2010). Cytogenetics of childhood acute myeloid leukemia: United Kingdom Medical Research Council treatment trials AML 10 and 12. J Clin Oncol.

[2] Shimoni A, Labopin M, Savani B, Volin L, Ehninger G, Kuball J (2016). Long-term survival and late events after allogeneic stem cell transplantation from HLA-matched siblings for acute myeloid leukemia with myeloablative compared to reduced-intensity conditioning: a report on behalf of the acute leukemia working party of European group for blood and marrow transplantation. J Hematol Oncol.

[3] Tomizawa D, Tawa A, Watanabe T, Saito AM, Kudo K, Taga T (2013). Excess treatment reduction including anthracyclines results in higher incidence of relapse in core binding factor acute myeloid leukemia in children. Leukemia..

[4] Gamis AS, Alonzo TA, Meshinchi S, Sung L, Gerbing RB, Raimondi SC (2014). Gemtuzumab ozogamicin in children and adolescents with de novo acute myeloid leukemia improves event-free survival by reducing relapse risk: results from the randomized phase III Children's oncology group trial AAML0531. J Clin Oncol.

[5] Rubnitz JE, Inaba H, Dahl G, Ribeiro RC, Bowman WP, Taub J (2010). Minimal residual disease-directed therapy for childhood acute myeloid leukaemia: results of the AML02 multicentre trial. Lancet Oncol.

[6] Willekens C, Blanchet O, Renneville A, Cornillet-Lefebvre P, Pautas C, Guieze R (2016). Prospective long-term minimal residual disease monitoring using RQ-PCR in RUNX1-RUNX1T1-positive acute myeloid leukemia: results of the French CBF-2006 trial. Haematologica..

[7] Yin JA, O'Brien MA, Hills RK, Daly SB, Wheatley K, Burnett AK (2012). Minimal residual disease monitoring by quantitative RT-PCR in core binding factor AML allows risk stratification and predicts relapse: results of the United Kingdom MRC AML-15 trial. Blood..

[8] Qin YZ, Wang Y, Xu LP, Zhang XH, Zhao XS, Liu KY (2020). Subgroup analysis can optimize the relapse-prediction cutoff value for WT1 expression after allogeneic hematologic stem cell transplantation in acute myeloid leukemia. J Mol Diagn.

[9] Huang XJ, Liu DH, Liu KY, Xu LP, Chen H, Han W (2006). Haploidentical hematopoietic stem cell transplantation without in vitro T-cell depletion for the treatment of hematological malignancies. Bone Marrow Transplant.

[10] Jourdan E, Boissel N, Chevret S, Delabesse E, Renneville A, Cornillet P (2013). Prospective evaluation of gene mutations and minimal residual disease in patients with core binding factor acute myeloid leukemia. Blood..

[11] Hollein A, Jeromin S, Meggendorfer M, Fasan A, Nadarajah N, Kern W (2018). Minimal residual disease (MRD) monitoring and mutational landscape in AML with RUNX1-RUNX1T1: a study on 134 patients. Leukemia..

[12] Zhu HH, Zhang XH, Qin YZ, Liu DH, Jiang H, Chen H (2013). MRD-directed risk stratification treatment may improve outcomes of t(8;21) AML in the first complete remission: results from the AML05 multicenter trial. Blood..

[13] Burnett AK, Hills RK, Milligan DW, Goldstone AH, Prentice AG, McMullin MF (2010). Attempts to optimize induction and consolidation treatment in acute myeloid leukemia: results of the MRC AML12 trial. J Clin Oncol.

[14] Hasle H, Kaspers GJ (2017). Strategies for reducing the treatment-related physical burden of childhood acute myeloid leukaemia - a review. Br J Haematol.

[15] Kim Y, Jang J, Hyun SY, Hwang D, Kim SJ, Kim JS (2013). Karyotypic change between diagnosis and relapse as a predictor of salvage therapy outcome in AML patients. Blood Res.

[16] Kurosawa S, Miyawaki S, Yamaguchi T, Kanamori H, Sakura T, Moriuchi Y (2013). Prognosis of patients with core binding factor acute myeloid leukemia after first relapse. Haematologica..

[17] Niewerth D, Creutzig U, Bierings MB, Kaspers GJ (2010). A review on allogeneic stem cell transplantation for newly diagnosed pediatric acute myeloid leukemia. Blood..

[18] Kaspers GJ, Zimmermann M, Reinhardt D, Gibson BE, Tamminga RY, Aleinikova O (2013). Improved outcome in pediatric relapsed acute myeloid leukemia: results of a randomized trial on liposomal daunorubicin by the international BFM study group. J Clin Oncol.

[19] Creutzig U, Zimmermann M, Dworzak MN, Gibson B, Tamminga R, Abrahamsson J (2014). The prognostic significance of early treatment response in pediatric relapsed acute myeloid leukemia: results of the international study relapsed AML 2001/01. Haematologica..

[20] Halaburda K, Labopin M, Mailhol A, Socie G, Craddock C, Aljurf M (2019). Allogeneic stem cell transplantation in second complete remission for core binding factor acute myeloid leukemia: a study from the acute leukemia working Party of the European Society for Blood and Marrow Transplantation. Haematologica.

[21] Campana D, Leung W (2013). Clinical significance of minimal residual disease in patients with acute leukaemia undergoing haematopoietic stem cell transplantation. Br J Haematol.

[22] Creutzig U, Zimmermann M, Bourquin JP, Dworzak MN, von Neuhoff C, Sander A (2011). Second induction with high-dose cytarabine and mitoxantrone: different impact on pediatric AML patients with t(8;21) and with inv(16). Blood..

[23] Klein K, Kaspers G, Harrison CJ, Beverloo HB, Reedijk A, Bongers M (2015). Clinical impact of additional cytogenetic aberrations, cKIT and RAS mutations, and treatment elements in pediatric t(8;21)-AML: results from an international retrospective study by the international Berlin-Frankfurt-Munster study group. J Clin Oncol.

[24] Boissel N, Leroy H, Brethon B, Philippe N, de Botton S, Auvrignon A (2006). Incidence and prognostic impact of c-kit, FLT3, and Ras gene mutations in core binding factor acute myeloid leukemia (CBF-AML). Leukemia..

[25] Manara E, Bisio V, Masetti R, Beqiri V, Rondelli R, Menna G (2014). Core-binding factor acute myeloid leukemia in pediatric patients enrolled in the AIEOP AML 2002/01 trial: screening and prognostic impact of c-KIT mutations. Leukemia..

[26] Lee JW, Kim S, Jang PS, Chung NG, Cho B, Im SA (2019). Prognostic role of Postinduction minimal residual disease and myeloid sarcoma type Extramedullary involvement in pediatric RUNX1-RUNX1T1 (+) acute myeloid leukemia. J Pediatr Hematol Oncol.

